# Chronic Nicotine Modifies Skeletal Muscle Na,K-ATPase Activity through Its Interaction with the Nicotinic Acetylcholine Receptor and Phospholemman

**DOI:** 10.1371/journal.pone.0033719

**Published:** 2012-03-19

**Authors:** Alexander V. Chibalin, Judith A. Heiny, Boubacar Benziane, Alexander V. Prokofiev, Alexander V. Vasiliev, Violetta V. Kravtsova, Igor I. Krivoi

**Affiliations:** 1 Department of Molecular Medicine and Surgery, Integrative Physiology, Karolinska Institutet, Stockholm, Sweden; 2 Department of Molecular and Cellular Physiology, University of Cincinnati, Cincinnati, Ohio, United States of America; 3 Department of General Physiology, St. Petersburg State University, St. Petersburg, Russia; University of Michigan, United States of America

## Abstract

Our previous finding that the muscle nicotinic acetylcholine receptor (nAChR) and the Na,K-ATPase interact as a regulatory complex to modulate Na,K-ATPase activity suggested that chronic, circulating nicotine may alter this interaction, with long-term changes in the membrane potential. To test this hypothesis, we chronically exposed rats to nicotine delivered orally for 21–31 days. Chronic nicotine produced a steady membrane depolarization of ∼3 mV in the diaphragm muscle, which resulted from a net change in electrogenic transport by the Na,K-ATPase α2 and α1 isoforms. Electrogenic transport by the α2 isoform increased (+1.8 mV) while the activity of the α1 isoform decreased (−4.4 mV). Protein expression of Na,K-ATPase α1 or α2 isoforms and the nAChR did not change; however, the content of α2 subunit in the plasma membrane decreased by 25%, indicating that its stimulated electrogenic transport is due to an increase in specific activity. The physical association between the nAChR, the Na,K-ATPase α1 or α2 subunits, and the regulatory subunit of the Na,K-ATPase, phospholemman (PLM), measured by co-immuno precipitation, was stable and unchanged. Chronic nicotine treatment activated PKCα/β2 and PKCδ and was accompanied by parallel increases in PLM phosphorylation at Ser^63^ and Ser^68^. Collectively, these results demonstrate that nicotine at chronic doses, acting through the nAChR-Na,K-ATPase complex, is able to modulate Na,K-ATPase activity in an isoform-specific manner and that the regulatory range includes both stimulation and inhibition of enzyme activity. Cholinergic modulation of Na,K-ATPase activity is achieved, in part, through activation of PKC and phosphorylation of PLM.

## Introduction

Acute exposure of skeletal muscles to low concentrations of acetylcholine (ACh, 100 nM) stimulates Na,K-ATPase electrogenic activity through a regulatory interaction between the muscle nicotinic acetylcholine receptor (nAChR) and the Na,K-ATPase [1], [2]. The nAChR specifically co- immunoprecipitates with both α1 and α2 isoforms of the Na,K-ATPase α-subunit and phospholemman (PLM), a muscle-specific auxiliary subunit of Na,K-ATPase which modulates enzyme activity [3], [4]. This suggests that these proteins assemble in a macromolecular complex capable of functional interactions. Acute stimulation of enzyme activity by ACh produces membrane hyperpolarization. Because the hyperpolarization occurs in the voltage range where Na^+^ channel slow inactivation is steeply voltage-dependent [5], it increases membrane excitability by shifting Na^+^ channels from the inactive to available conformation. Skeletal muscles are normally exposed to nanomolar concentrations of ACh for some time following nerve excitation, after the larger bolus of ACh has been hydrolyzed by acetylcholinesterase. Therefore, when a quiescent muscle is stimulated by nerve input, this ACh-induced hyperpolarization primes the muscle to respond to an increased level of nerve activity.

The hyperpolarizing effect of acute exposure to ACh is specific for the Na,K-ATPase α2 isoform and is most likely mediated by a desensitized state of the nAChR [1], [2]. Acute nanomolar concentrations of nicotine, an exogenous non-hydrolyzable nAChR agonist, also stimulate the Na,K-ATPase α2 isoform by this mechanism [1], [2], [6]. This finding suggested that chronic *in vivo* exposure to nicotine, which reaches hundreds of nM up to µM levels during tobacco use [7], [8], might produce long-term effects on the Na,K-ATPase and membrane potentials in skeletal muscle. This question has not been investigated previously. It is commonly thought that muscle nAChRs are largely spared the effects of nicotine use because the affinity of the muscle nAChR for nicotine is significantly lower than that of brain nAChRs [9]. However, chronic agonist exposure promotes nAChR desensitization [10], and the desensitized state of the nAChR is the favored conformation which interacts with the Na,K-ATPase [1], [2]. In addition, the stable association of PLM with the Na,K-ATPase isoforms suggested that this regulatory interaction may modulate enzyme activity through a kinase-dependent phosphorylation of PLM. In cardiac and smooth muscle cells, phosphorylation of PLM at Ser^63^ and Ser^68^ by PKC stimulates Na,K-ATPase α2 activity by relieving an inhibitory interaction of PLM with the enzyme [11]. The role of PLM in regulating Na,K-ATPase activity in skeletal muscle is not known and the significance of its association with the nAChR- Na,K-ATPase complex has not been previously investigated.

This study examines the consequences of chronic nicotine exposure on membrane potentials and the activity of the Na,K-ATPase α1 and α2 isoforms in skeletal muscle, and examines whether regulation of enzyme activity by the nAChR–Na,K-ATPase complex involves phosphorylation of PLM by PKC. We administered nicotine orally to rats for 21–31 days and analyzed its effect on the resting membrane potential (RMP) of diaphragm skeletal muscle; the electrogenic transport activity of the Na,K-ATPase α1 and α2 isoforms; expression of the Na,K-ATPase α isoforms, the nAChR and PLM; the stability of the nAChR-Na,K-ATPase-PLM complex measured by co-immunoprecipitation; activation of PKCα/β2 and PKCδ, phosphorylation of PLM at Ser^63^ and Ser^68^, and the plasma membrane content of Na,K-ATPase. The majority of these assays was made using tissue from the same muscles used to measure electrogenic activity in order to directly relate changes in Na,K-ATPase activity to these measures.

Our results further demonstrate that the nAChR interacts with the Na,K-ATPase to modulate enzyme activity and that both Na,K-ATPase α1 and α2 isoforms can be regulated by this interaction in an isoform-specific manner. The regulatory range includes both stimulation and inhibition of enzyme activity. The same nicotine treatment activates PKC and increases PLM phosphorylation, suggesting that cholinergic modulation of Na,K-ATPase activity may utilize this regulatory pathway.

## Materials and Methods

### Materials

Ouabain, nicotine ((−)nicotine hydrogen tartrate) and other chemicals were from Sigma. Specific monoclonal antibodies against the Na,K-ATPase α1- and α2-subunits were generously provided by Dr. M. Caplan (Yale University, New Haven, CT) and Dr. K. Sweadner (Boston, MA). The antibodies against total PLM were acquired from the ProteinTech Group (Chicago, IL). Antibodies against phosphorylated PLM Ser^63^, and Ser^68^ were kindly donated by Dr. J. Cheung (Thomas Jefferson University, Philadelphia, PA). Rabbit polyclonal antibodies against anti-phospho PKCα/β, δ and anti- PKC α, β, δ were from Cell Signaling Technology, Inc (Beverly, MA). The antibody against the nAChRα1 subunit was from Abcam. Horseradish peroxidase-conjugated goat anti-rabbit and anti-mouse IgG were from Bio-Rad. Reagents for enhanced chemi luminescence (ECL) were from Amersham Pharmacia Biotech. All other reagents were analytical grade.

### Animals

Chronic nicotine exposure, electrophysiological experiments and biochemical assays were performed using adult male Wistar rats (180–200 g). The rats were anesthetized (ether) and euthanized by cervical dislocation, and the diaphragm muscle with intact tendons was removed.

### Ethics Statement

This study was carried out in accordance with the recommendations in the Guide for the Care and Use of Laboratory Animals of the National Institutes of Health. The protocol was approved by the Ethics Committee of St. Petersburg State University and the National Ministry of Health (Approval 19.06.2003/267) of the Russian Federation. All surgery was performed under anesthesia (ether), and all efforts were made to minimize suffering.

### Chronic nicotine treatment

Nicotine was administered orally in the drinking water at a concentration of 60 mg/l, using standard protocols [12]–[14] which correspond to a dose of 2–4 mg/kg per day. This approach has been shown to produce a plasma nicotine pattern similar to that seen in smokers [13], [15]. The drinking water was the sole source of fluid and also contained 2% saccharin. Typically, two rats per day (from control and nicotine groups) were used starting on day 21, while the remaining rats continued to receive nicotine and were used for up to 31 days. The oral protocol delivered nicotine in a cyclical manner with transient increases, which more closely reproduces the condition of human tobacco use. At the end of treatment, two hemi-diaphragms were dissected from each rat. Strips from the left hemi-diaphragm muscle were used immediately for membrane potential measurements and the remaining tissue was quickly frozen in liquid nitrogen for later biochemical assays.

### Membrane potential recording

The experiments were performed on freshly isolated rat diaphragm. A 10–15 mm wide diaphragm strip with nerve stump was placed in a 2-ml Plexiglas chamber. The chamber was continuously perfused with a physiological solution containing (mM): NaCl, 137; KCl, 5; CaCl_2_, 2; MgCl_2_, 2; NaHCO_3_, 24; NaH_2_PO_4_, 1; glucose, 11; pH 7.4. The solution was continuously bubbled with 95% O_2_ and 5% CO_2_ gas mixture and maintained at 28°C. The muscle was equilibrated for 1 hour prior to the start of recording. RMPs were recorded from extra junctional membrane regions using intracellular microelectrodes, as described previously [1], [2]. RMPs were recorded from 25–35 different fibers within each muscle, over a total recording time of about 5–10 min. The entire protocol was repeated in muscles from different animals.

### Measurement of Na,K-ATPase electrogenic activity in intact muscle

Na,K-ATPase transport activity was determined in intact skeletal muscle fibers by measuring the ouabain-sensitive change in membrane potential. This change is generated by electrogenic Na,K-ATPase transport and is a sensitive, real-time assay of Na,K-ATPase activity in intact muscle cells [16].

### Muscle membrane preparation

Approximately 50 mg of rat diaphragm muscle was weighed, minced, and homogenized with a Polytron at low speed (setting 4, 2×10 sec) in a buffer containing: 20 mM Tris-HCl, 250 mM sucrose, 1 mM EDTA, 1 µM okadaic acid, 1 mM phenylmethylsulfonyl fluoride (PMSF), and 10 µg/ml each of aprotinin, leupeptin, and pepstatin. The resulting homogenate was centrifuged for 10 min at 3,000×g. The supernatant was collected and kept on ice. The pellet was re-suspended in homogenization buffer and centrifuged again for 10 min at 3,000×g. The supernatants were pooled, aliquoted, and stored at −70°C. A purified plasma membrane fraction was prepared from rat diaphragm muscle using a step sucrose gradient as described previously [17].

### Western Blot

Western blots were performed as described [2]. In brief, aliquots of muscle homogenate (20 µg) or plasma membrane fraction (2.5 µg) were re-suspended in Laemmli sample buffer. The proteins were separated by SDS/PAGE, transferred to polyvinylidenedifluoride (PVDF) membranes (Millipore, MA), blocked with 7.5% non-fat milk, washed with TBST (10 mM Tris HCl, 100 mM NaCl, 0.02% Tween 20) and incubated with the appropriate primary antibodies overnight at 4°C. Membranes were washed with TBST and incubated with the appropriate secondary antibody. Proteins were visualized by enhanced chemi luminescence (ECL) and quantified by densitometry. Ponceau S staining was used to verify equal gel loading [18] and for normalization.

### Co-immunoprecipitation assays

Co-immunoprecipitation assays were carried out as described previously [2]. Briefly, muscles were solubilized with lysis buffer (137 mM NaCl, 2.7 mM KCl, 1 mM MgCl_2_, 20 mM Tris, pH 8.0, 1% Triton X-100, 10% (v/v) glycerol, 10 mM NaF, 0.5 mM Na_3_VO_4_, 5 µg/ml leupeptin, 0.2 mM phenylmethylsulfonyl fluoride, 5 µg/ml aprotinin, and 1 µM microcystin). Immunoprecipitation was carried out using a primary antibody to the nAChRα1 subunit (Abcam ab11149) followed by affinity purification using protein G-agarose beads (Dynal). After incubation with protein G-agarose beads for 1 h at room temperature, the immuno complex was washed in lysis buffer followed by PBS. The protein samples were probed by Western Blot with primary antibodies and horseradish peroxidase-conjugated secondary antibody. The proteins were visualized by ECL and quantified by densitometry.

### [^3^H]ouabain binding in control and PMA treated intact skeletal muscles

A rat soleus muscle was dissected and equilibrated for 30 min in standard Krebs-Ringer, then incubated in K^+^-free Krebs–Ringer buffer containing 100 nM PMA and 2 µM [^3^H]ouabain (0.5 µCi ml^−1^) for 0, 30, 60 and 120 min, followed by 4×15 min washouts in ice-cold K^+^-free Krebs–Ringer buffer. Following washout the muscles were frozen and divided. One half of the sample was soaked in 0.3 M trichloroacetic acid (TCA) and taken for counting activity. The content of [^3^H] binding sites was expressed as picomoles per gram wet weight. The remainder was analyzed by Western Blot for PKC activation and PLM phosphorylation using isoform-specific antibodies.

### Data analysis

Data are given as the mean ± SEM. Statistical significance of the difference between groups means (control vs. nicotine-treated) was evaluated using a Student's t-test (ORIGIN 6.1. software). The distribution of RMPs was fitted to a Gaussian function to obtain the mean RMP of each group. Normality of the distribution was tested using the Kolmogorov-Smirnov test with Dallal-Wilkinson-Lillifors correction (GraphPad Prism 5).

## Results

### Chronic nicotine exposure depolarizes the resting membrane potential of rat diaphragm

**Figure 1:**
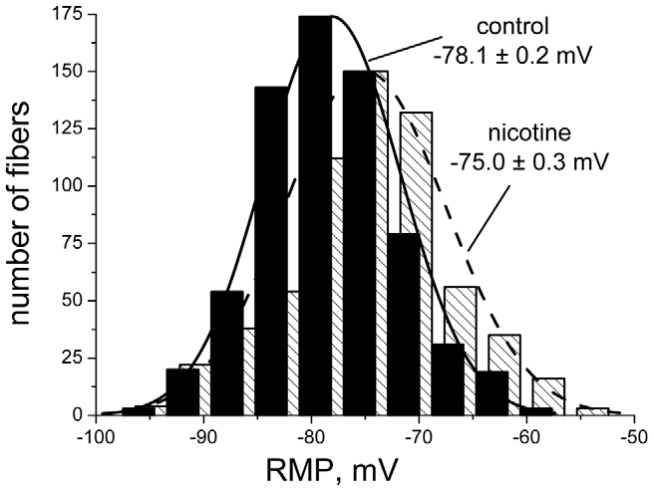
Distribution histogram of resting membrane potentials in the diaphragm of control (solid bars) and nicotine-treated rats (striped bars). Treated animals received nicotine orally in the drinking water for 21–31 days prior to tissue removal. RMPs were recorded from 622 fibers from 9 muscles (nicotine) and 676 fibers from 10 control muscles (vehicle). The solid and dashed curves are Gaussian fits to the RMP distribution for each group. The distribution of RMPs in each group was consistent with a normal distribution based on the Kolmogorov-Smirnov normality test (Methods). The classes on the histograms are grouped (using ORIGIN 6.1) with Bin size 4.1 mV for 12 bins, in the range from −50 mV to −97.5 mV. For ease of visualization, the gap between bars was chosen = 0, overlap is 60%.

Chronic oral nicotine exposure depolarized skeletal muscles by +3.1±0.4 mV (p<0.01) compared to paired controls (**Fig. 1**). RMPs of both control and nicotine-treated muscles showed a Gaussian distribution, reflecting the typical range of RMPs present in different fibers within a muscle. Chronic nicotine produced a simple shift of the mean RMP without change in normal distribution.

### The depolarization produced by chronic nicotine exposure results from decreased electrogenic transport by the Na, K-ATPase

**Figure 2:**
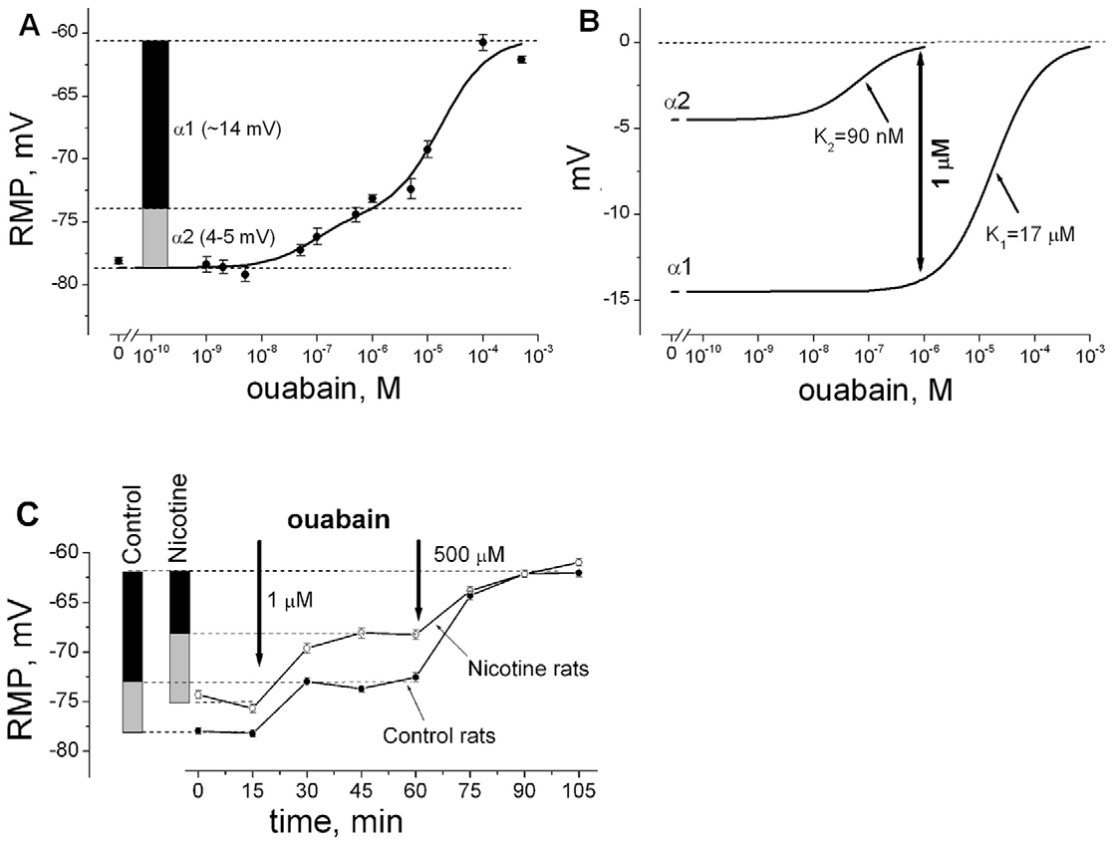
Contributions to the resting membrane potential (mV) from electrogenic active transport by the α1 and α2 Na, K-ATPase isozymes in the diaphragm muscle of control and chronic nicotine-exposed rats. **A**) RMP of muscle fibers versus ouabain concentration. Each data point represents the mean ± SEM of 130–170 measurements from 4–6 muscles. The solid line is a nonlinear regression fit to a two-site binding model: RMP = RMP_0_+A_1_/(1+[I]/K_1_)+A_2_/(1+[I]/K_2_), where RMP_0_ is the RMP when both ouabain-binding sites are inhibited; K_1_ and K_2_ are the half maximal ouabain concentrations for ouabain binding to α1 and α2 isoforms, respectively; A_1_ and A_2_ (mV) are their respective contributions to the RMP and [I] is the inhibitor (ouabain) concentration. The left vertical bar indicates the electrogenic potentials contributed by the α1 (black) and α2 (grey) isoforms obtained from the fitted data. Horizontal dashed lines show the predicted RMP levels for three cases: when both α isoforms are inactive (∼−61 mV, E_Nernst_ alone), when only α1 is active (∼−74 mV), and when both α1 and α2 are active (∼−78 mV). Muscles were incubated with the indicated concentration of ouabain for one hour before the start of recording. **B**) Concentration-dependence and K values for inhibition of the α2 and α1 isozymes, computed from the data in panel A. **C**) Changes in RMP elicited by 1 µM and 500 µM ouabain in the diaphragm of control (filled circles) and nicotine-treated (open circles) rats. Rats received nicotine orally for 21–31 days, as described in Methods. Measurements are from the same muscles as in Fig. 1 (oral nicotine). Arrows indicate when ouabain was added and the horizontal bar indicates when ouabain was present in the solution. RMPs were measured 15, 30 and 45 minutes and stabilized to a new level within 30 min of each solution change. Left vertical bars denote the electrogenic potentials contributed by the α1 (black) and α2 (grey) isozymes. Measurements are from 10 (control) and 9 (nicotine-treated) animals.

Our previous finding that acute nicotine exposure at nM concentrations, acting through the nAChR, can modulate the activity of the Na,K-ATPase α2 isoform [1], [2], [6] led us to investigate whether the depolarization produced by chronic nicotine is mediated by this same interaction. Skeletal muscles express two isoforms of the Na,K-ATPase, α1 and α2 [19]. **Figs. 2A & 2B** show the method used to measure the basal transport activity of each isoform in intact muscle from its electrogenic contribution to the RMP. The method is based on the more than 100-fold difference in affinities of the rodent α1 and α2 Na,K-ATPase isoforms for ouabain. The Na,K-ATPase is the only known receptor for ouabain. Active transport by the Na,K-ATPase generates a negative membrane potential, V_pump_, due to the net outward transfer of one positive charge per transport cycle (3 Na^+^ out per 2 K^+^ in). V_pump_ adds directly to the Nernst potential arising from the ion concentration differences (E_Nernst_) and brings the RMP to a more negative value than expected from the ion gradients alone (RMP = E_Nernst_+V_pump_) [20]. This ouabain-inhibitable, negative component of the RMP directly reports the resting transport activity of the Na,K-ATPase in intact muscle fibers. V_pump_ is large in skeletal muscles and hyperpolarizes the membrane by −15 to −18 mV (compared to only a few mV in nerve and other cell types). The ouabain concentration-dependence of V_pump_ was best fit to a two-site binding model (smooth curve and legend) with ouabain affinities of 17 µM and 90 nM (**Fig. 2A, B**) for the rat α1 and α2 isoforms, respectively. These affinities correspond closely to measurements in other tissues and membrane preparations using ouabain binding assays [21], [22]. Based on this analysis, we used ouabain concentrations of 1 µM and 500 µM to separate the electrogenic contributions of α2 and α1 to the RMP. Ouabain at 1 µM inhibits more than 95% of α2 activity while leaving α1 activity unchanged; 500 µM ouabain completely inhibits both isoforms (**Fig. 2B**).

In control muscles, total electrogenic activity by both isoforms contributes −18 mV to the RMP. Of this, the α1 isoform generates −13.9±1.4 mV and the α2 isoform generates −4.4±1.4 mV (black and grey bars, respectively). Therefore, the α1 and α2 isoforms contribute 75% and 25%, respectively, of the basal Na/K transport required to maintain ion gradients and the RMP. Chronic nicotine treatment alters these contributions in an α-isoform dependent manner (**Fig. 2C**). In control muscle fibers (sham-treated animals, filled circles), electrogenic transport by the Na,K-ATPase α1 generated −11.0±0.4 mV, and the α2 isoform generated −5.0±0.4 mV. Both values are close to those in reference (untreated) animals (**Fig. 2A**). Chronic nicotine treatment (open circles) significantly *de*creased the electrogenic potential contributed by α1 activity to −6.6±0.5 mV (−4.4 mV, p<0.001); and it *in*creased the electrogenic potential contributed by α2 activity to −6.8±0.5 mV (+1.8 mV, p<0.01). Overall, chronic nicotine treatment decreased the resting transport activity of the α1 isozyme by 60% and stimulated the resting transport activity of α2 by 36% (**Table 1**). These combined actions produced a net decrease in total V_pump_ of −2.6 mV (p<0.001) and net depolarization. Again, these measurements were made in diaphragm skeletal muscles from the same paired animals used for **Fig. 1** (oral nicotine treatment, 21–31 days), which allowed us to directly relate the depolarization to a net loss of electrogenic Na,K-ATPase activity.

**Table 1:** Mean RMPs in the diaphragm muscle of control and chronic nicotine-treated rats, and the electrogenic potentials generated by α1 and α2 Na,K-ATPase basal transport.

	Initial RMP	RMP in 1 µM ouabain	RMP in 500 µM ouabain	Electrogenic potential generated by α2	Electrogenic potential generated by α1	Total electrogenic potential (V_pump_)
	*mV*	*mV*	*mV*	*mV*	*mV*	*mV*
Control	−78.1±0.2 (n = 676)	−73.1±0.3 (n = 683)	−62.1±0.3 (n = 691)	−5.0±0.4	−11.0±0.4	−16.0±0.4
Chronic nicotine	−75.0±0.3*** (n = 622)	−68.2±0.4*** (n = 625)	−61.6±0.3 (n = 618)	−6.8±0.5**	−6.6±0.5***	−13.4±0.4***

RMPs were computed from measurements in muscles perfused sequentially with no ouabain (control solution), 1 µM ouabain, or 500 µM ouabain, as shown in Fig. 2C.

** p<0.01 and

*** p<0.001, compared to control. Treated rats received nicotine orally for 21–31 days prior to tissue removal. RMPs were measured 30–45 min after each solution change. n = number of fibers. Mean RMPs were obtained from a fit of the RMPs in each group to a Gaussian function, after confirming that the RMPs distributed normally (Kolmogorov-Smirnov test, Methods).

Importantly, when all Na,K-ATPase electrogenic activity is inhibited, the RMPs of both control and nicotine-treated muscles stabilize at the same value of −62 mV. This result confirms that chronic nicotine treatment specifically alters Na,K-ATPase activity without changing the Nernst potential which, in the absence of electrogenic transport, is determined solely by the membrane permeability and ion gradients (Goldman-Hodgkin-Katz equation).

### Chronic nicotine treatment does not alter the muscle content of nAChR, Na,K-ATPase α1 or α2 subunits, but decreases α2 content in the sarcolemma

**Figure 3:**
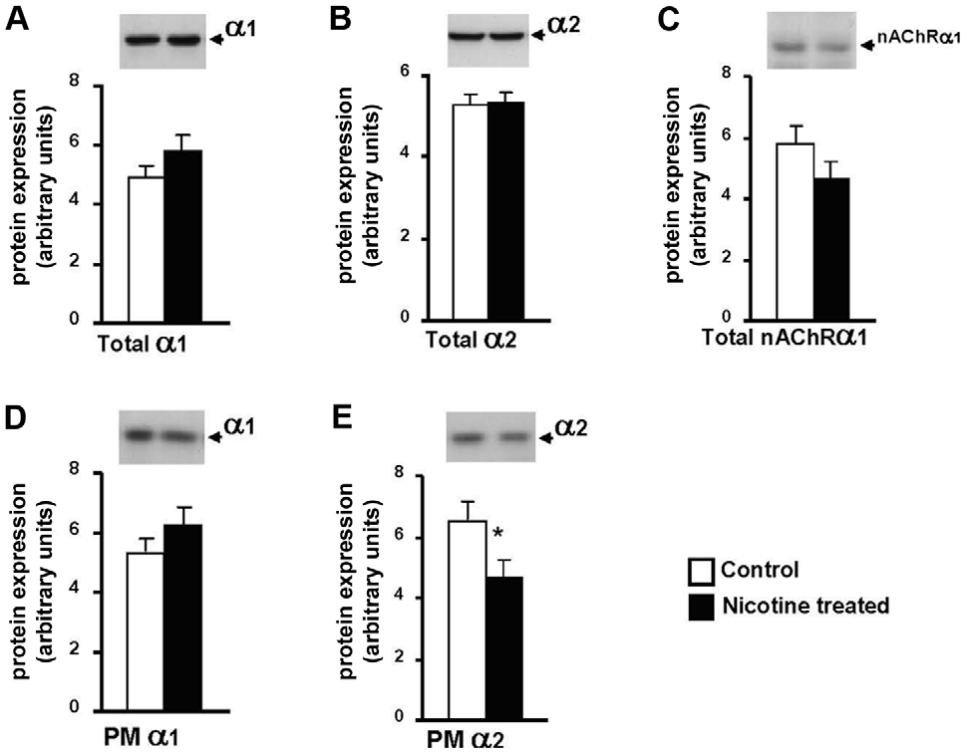
Na,K-ATPase α1 and α2 and nAChR content in diaphragm muscles of control and nicotine-treated rats. **A**, **B**, **C** – whole homogenate; **D**, **E** – plasma membrane fraction. Upper panels show representative immunoblots; lower panels show mean densities ± SE from 9–10 blots prepared using different muscle samples. * p<0.05. Nicotine was administered orally for 21–31 days as described in Methods. Assays were made using diaphragm tissue from the same muscles used for RMP and activity measurements (Fig. 1 & 2C, oral nicotine).

It is possible that chronic nicotine exposure may decrease total pump activity by altering the expression of Na,K-ATPase α1 or α2 subunits, or the nAChR. To address this question, we measured the content of Na,K-ATPase α subunits and the nAChR before and after chronic nicotine treatment (**Fig. 3**). Chronic nicotine treatment did not change total Na,K-ATPase α1 or α2 or nAChRα1 content measured in whole homogenates from skeletal muscle (**Fig. 3**). In a purified sarcolemmal fraction, there was also no change in Na,K-ATPase α1 expression. However, the plasma membrane content of α2 decreased by 25% (p<0.05), suggesting that chronic nicotine may alter the distribution of the α2 isozyme between an intracellular pool and the sarcolemmal. This change is opposite in direction to its increased electrogenic activity which, therefore, must arise from higher specific activity of α2 enzyme in the sarcolemma. These results indicate that the increased α2 and decreased α1 electrogenic activity produced by chronic nicotine exposure are not explained by altered expression of Na,K-ATPase α subunits or the nAChR.

### Chronic nicotine treatment does not alter the molecular interaction between the nAChR and the Na,K-ATPase alpha subunits and PLM

**Figure 4:**
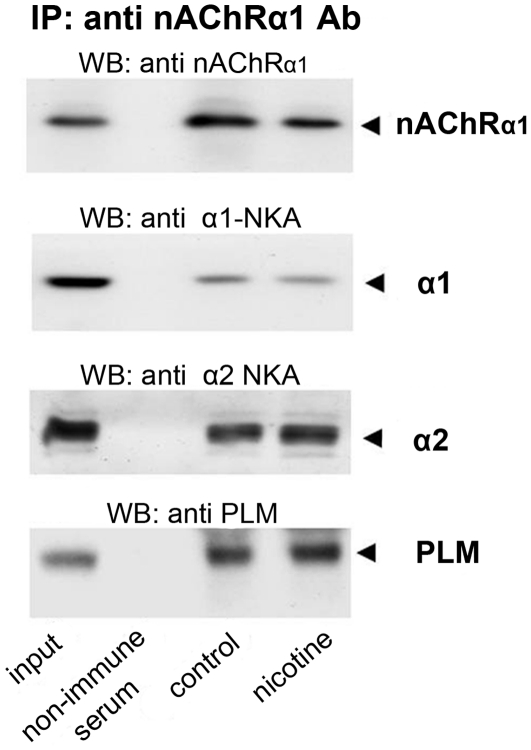
The nAChRα1 subunit and the Na,K-ATPase α1 and α2 subunits and PLM co-immunoprecipitate in rat diaphragm muscle after 21–31days of sham or oral nicotine treatment. Skeletal muscle protein was prepared from control and nicotine treated animals and immunoprecipitated (*IP*) with monoclonal antibodies against the nAChRα1 subunit. Precipitates were probed by Western blot (*WB*) using antibodies against the nAChRα1, Na,K-ATPase α1 and α2, and PLM. A positive control (*lane 1*, input) confirmed the presence of each species in the control sample before IP. Each panel is a representative Western blot from 7–8 independent experiments. Protein homogenates were prepared using diaphragm tissue from the same muscles used for RMP and activity measurements (Fig. 1 & 2C, oral nicotine).

The current finding that chronic exposure to nicotine can also modify Na,K-ATPase activity and the RMP suggested that chronic nicotine may work through the same nAChR-Na,K-ATPase complex which mediates the previously described acute effects of nicotinic agonists on enzyme activity [2]. Therefore, we examined whether the chronic nicotine treatment alters the physical association between the nAChRα1 subunit, the Na,K-ATPase α1 or α2 subunits and PLM. Our results (**Fig. 4**) confirm that the nAChR and the Na,K-ATPase α1 or α2 subunits and PLM co-immunoprecipitate in rat diaphragm and that their association is retained through 21–31 days of sham or oral nicotine treatment. Therefore, the effects of chronic nicotine exposure on Na,K-ATPase α1 or α2 activity are not due to disruption of the nAChR-Na,K-ATPase-PLM association, which remains stable and capable of interactions.

### Chronic nicotine treatment activates PKC and promotes PLM phosphorylation

**Figure 5:**
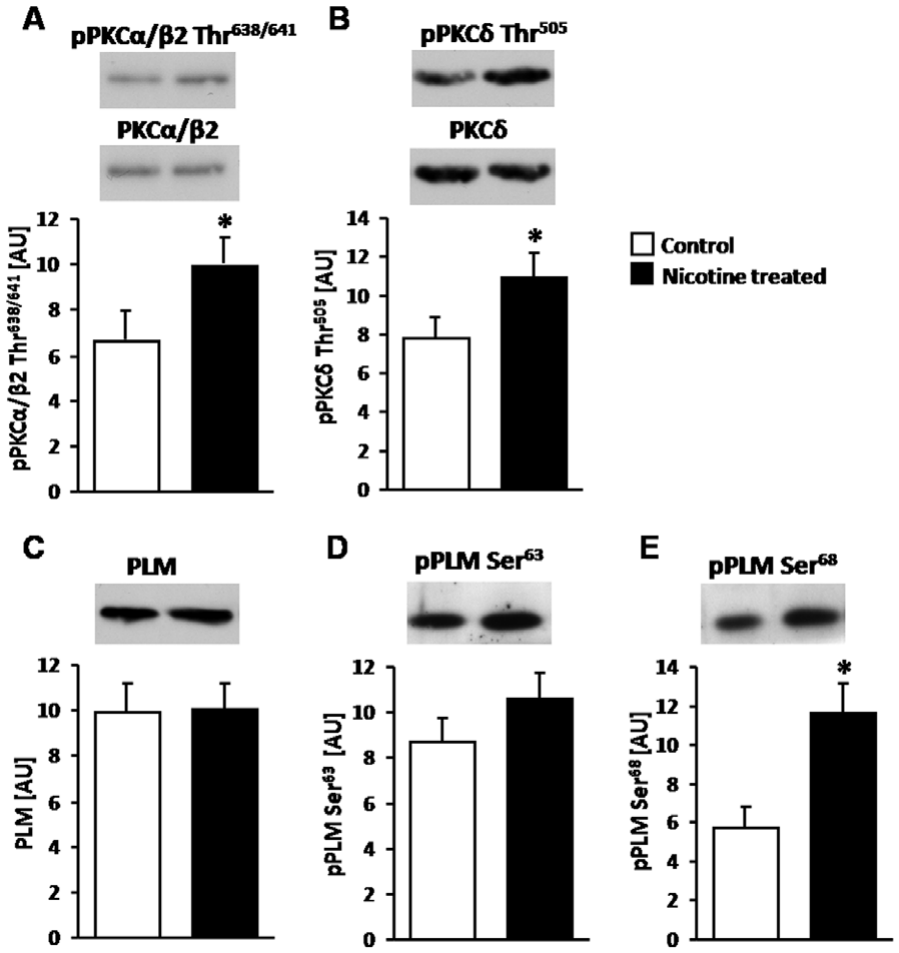
Chronic nicotine treatment activates PKCα/β2 (A) and PKCδ (B) and increases PLM phosphorylation at Ser^63^ (D) and Ser^68^ (E). Total PKCα/β2 (**A**), PKCδ (**B**), or PLM (**C**) abundance was not affected by the nicotine treatment. Bar graphs show the mean density from 8–9 measurements. A representative Western Blot is shown above each graph. Blots were probed with specific antibodies to activated PKCα/β2 (PKCα/β2 Thr^638/641^) and total PKCα/β2, activated PKCδ (PKCδ Thr^505^) and total PKCδ, total PLM or PLM phosphorylated at Ser^63^ (pPLM Ser^63^) or Ser^68^ (pPLM Ser^68^). Protein homogenates were prepared from the same samples used for RMP and activity measurements, obtained from diaphragm muscles of rats after 21–31 day treatment with oral nicotine or sham (control) (Fig. 1 & 2C). * p<0.05. Y-axis, arbitrary units (AU).

PLM (FXYD1) is one of the most abundant phospho proteins in skeletal and cardiac muscle. It is a member of the FXYD family of small, single membrane-spanning proteins which act as tissue-specific regulators of the Na,K-ATPase [23]. Phosphorylation of PLM by PKA and PKC alters the enzyme's substrate affinity or turnover in a cell- and Na,K-ATPase isoform-specific manner [3], [24]. PLM associates with the Na,K-ATPase α1 and α2 isoforms in skeletal and cardiac muscle [11], [25], [26]. In cardiac myocytes, phosphorylation of PLM by PKC (at Ser^63^ or Ser^68^) increases the transport activity of the α2 isoform (but not α1) by relieving an inhibitory interaction of PLM with the enzyme [11]. In smooth muscle, PKC mediated phosphorylation of PLM occurs only when it is associated with the α2 isoform, and leads to increased enzyme activity [27]. The role of PLM phosphorylation by PKC on Na,K-ATPase activity in skeletal muscle and the significance of its association with the nAChR-Na,K-ATPase complex is not known. Therefore, we examined whether PKC activation and PLM phosphorylation play a role in the stimulation of specific Na,K-ATPase activity by nicotine. Our results show that the same chronic oral nicotine treatment which alters Na,K-ATPase electrogenic activity also activates PKCα/β2 (**Fig. 5A**) and PKCδ (**Fig. 5B**), without change in total PKCα/β2 or PKCδ content. In parallel, chronic nicotine increases PLM phosphorylation without change in total PLM content (**5C,D, E**). Phosphorylation at Ser^68^ increased 2-fold (**5E**; p<0.01), while phosphorylation of Ser^63^ tended to increase (p = 0.08). This result demonstrates that nicotine, a specific agonist of the nAChR, is able to activate PKC and induce phosphorylation of PLM. It supports the idea that PLM phosphorylation by PKC may play a role in the modulation of Na,K-ATPase α2 activity by nicotine acting through the nAChR.

**Figure 6:**
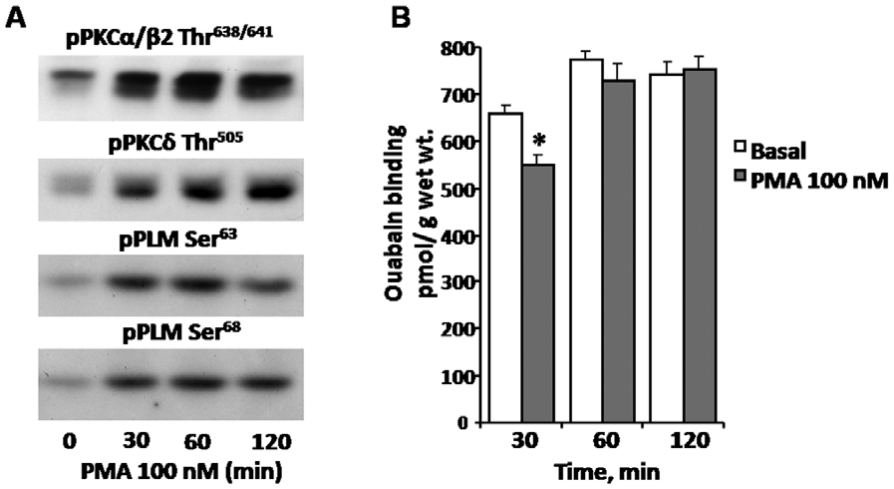
Effects of PMA on PKC and PLM phosphorylation and [^3^H]ouabain binding to intact rat skeletal muscle. **A**) Activation of PKCα/β2 and PKCδ by PMA (phorbol-12-myristate-13-acetate, 100 nM) induces parallel increases in PLM phosphorylation at Ser^63^ and Ser^68^. A rat soleus muscle was dissected and equilibrated for 30 min in standard Krebs-Ringer solution, then incubated in K^+^-free Krebs–Ringer buffer containing 100 nM PMA and 2 µM [^3^H]ouabain for 0, 30, 60 and 120 min, followed by 4×15 min washout in ice-cold K^+^-free Krebs–Ringer buffer. Five independent experiments were performed and a representative Western blot is shown. **B**) [^3^H]ouabain binding site content in intact rat soleus muscle was determined directly in the same experiment and expressed per gram wet weight. Mean values ±S.E.M. are shown, * p<0.05, *n* = 5.

To further investigate the mechanism by which nicotine acting through the nAChR-Na,K-ATPase complex regulates Na,K-ATPase activity, we examined the relationship between PKC activation, PLM phosphorylation and [^3^H]ouabain (2 µM) binding, which reflects α2 Na,K-ATPase content in the sarcolemma (**Fig. 6**). Acute activation of PKC by PMA (100 nM) increases PLM phosphorylation at Ser^63^ and Ser^68^ (**Fig. 6A**), similar to the effect of chronic nicotine. PKC activation and PLM phosphorylation follow a parallel time course; both are stimulated within 30 minutes and the changes are sustained for up to 120 min. The same treatment (100 nM PMA) applied to intact rat skeletal muscle does not change the content of α2 Na,K-ATPase in the plasma membrane (**Fig. 6B**). Over the same time period, ouabain binding to the Na,K-ATPase reached equilibrium and there was no difference in the maximum amount of ouabain bound between control and PMA treated. [^3^H]ouabain binding to intact skeletal muscle measures only Na,K-ATPase pumps in the plasma membrane which have the extracellular ouabain binding site accessible to ligand. This result suggests that acutely activated PKC stimulates Na,K-ATPase specific activity by a mechanism which includes PLM phosphorylation, without change in the total content of Na,K-ATPase in the plasma membrane.

## Discussion

### Chronic nicotine exposure alters electrogenic Na,K-ATPase activity in an isoform-specific manner

Previous studies have established that the skeletal muscle nAChR and the Na,K-ATPase associate as a functional complex to modulate Na,K-ATPase activity, and thereby the membrane potential [1], [2]. This complex includes, at a minimum, the nAChR α1, the Na,K-ATPase α1 or α2 subunits, PLM, and caveolin-3 [2]. Acute, nanomolar concentrations of nicotinic agonists act through the nAChR to stimulate Na,K-ATPase electrogenic transport, causing membrane hyperpolarization. This acute action of nicotinic agonists is selective for the Na,K-ATPase α2 isoform [1], [2], [6]; it does not alter the transport activity of the Na,K-ATPase α1 isoform. Because steady, nanomolar concentrations of ACh are normally present in the postsynaptic neuromuscular junction during nerve activity, this interaction is expected to enhance the safety factor of neuromuscular transmission and muscle excitability, especially during high-frequency electrical activity [5], [28].

The present study demonstrates that nicotine at chronic doses, acting through the same nAChR-Na,K-ATPase complex, is also able to modulate Na,K-ATPase activity. Moreover, the effects of chronic nicotine are isoform-specific and include both stimulation and inhibition of enzyme activity. Chronic nicotine stimulates the Na,K-ATPase α2 isoform (+1.8 mV increase in electrogenic potential), similar to the action of acute nicotine. However, chronic nicotine *inhibits* the α1 isoform (−4.4 mV decrease). The combined action is a net *de*crease in total Na,K-ATPase activity and membrane *de*polarization (∼−3 mV). The depolarization is blocked by ouabain, indicating that it results from reduced electrogenic transport by the Na,K-ATPase. This signature profile — induction by a specific nAChR agonist and inhibition by a highly specific Na,K-ATPase antagonist — indicates that the depolarization is mediated by the functional interaction between the nAChR and the Na,K-ATPase. Thus, while both acute and chronic levels of nicotine, acting through the nAChR–Na,K-ATPase complex, stimulate the α2 isoform, chronic nicotine levels produce a net decrease in Na,K-ATPase activity. These new findings demonstrate that the nAChR is capable of functional interactions with both the α1 and α2 Na,K-ATPase isoforms, and that the regulatory range includes both inhibition and stimulation of enzyme transport. An interaction of the nAChR with both Na,K-ATPase isoforms is consistent with our finding that the nAChR co-immuno precipitates with both α1 and α2 subunits ([2] and **Fig. 4**).

### Chronic nicotine-induced depolarization is not due to altered expression of Na,K-ATPase α subunits, nAChR, or their association with each other and PLM

Neither the increased activity of the α2 isoform nor the decreased activity of α1 is explained by altered expression of Na,K-ATPase α subunits or the nAChR. Moreover, chronic nicotine does not alter the physical association between the nAChR and the Na,K-ATPase α1 or α2 isoforms and PLM. Their association is stable and remains capable of functional interactions during chronic nicotine treatment. The decrease (25%) in Na,K-ATPase α2 content in the sarcolemma without change in total homogenate suggests that chronic nicotine exposure may alter its targeting to the sarcolemma, without change in synthesis or degradation. This result excludes the possibility that the increase in Na,K-ATPase α2 activity is due to increased expression. It suggests that the stimulation of Na,K-ATPase α2 activity by chronic nicotine results from increased specific activity of sarcolemmal enzyme.

This effect of chronic nicotine on sarcolemmal Na,K-ATPase α2 content in skeletal muscle is different from that in brain tissues. Na,K-ATPase α2 content decreases significantly in whole homogenates of cerebral micro vessels and brain tissues of rats exposed to chronic nicotine [29], and the decrease is associated with a modest decrease in Na,K-ATPase activity. These differences may reflect tissue-specific regulatory mechanisms. The effects of chronic nicotine on intact muscle differ also from the effects of carbamylcholine on cultured C2C12 cells. Chronic exposure of cultured C2C12 myotubes to micromolar carbamylcholine for 3 days enhances electrogenic transport by the Na,K-ATPase, causing membrane hyperpolarization [30], [31]. The hyperpolarization was attributed to increased abundance of the α2 isoform, possibly interacting with nAChRs.

### Role of PKC activation and PLM phosphorylation in regulation of Na,K-ATPase activity by the nAChR

PLM is a muscle specific auxiliary subunit of the Na,K-ATPase which modulates enzyme activity [3], [4], [24]. In cardiac and smooth muscle, phosphorylation of PLM at Ser^63^ and Ser^68^ by PKC stimulates Na,K-ATPase α2 activity by relieving an inhibitory interaction of PLM with the enzyme [11]. The role of PLM in regulating the Na,K-ATPase activity in skeletal muscle is less well understood, and the significance of the association of nAChRs with the Na,K-ATPase and PLM has not been previously investigated. Our present results demonstrate that chronic nicotine exposure activates PKC and promotes phosphorylation of PLM at Ser^63^ and Ser^68^, without altering PLM content or its association with the Na,K-ATPase. This finding identifies nicotine (and presumably other nicotinic agonists) as an activator of PKC in skeletal muscle.

It is possible that additional signaling partners may participate in this regulation. The full complement of proteins in the complex which includes the muscle nAChR and Na,K-ATPase is not known. The proteome of other nAChR subtypes includes PKA, PKC, other kinases and phosphatases, and G-proteins (nAChRα7; ‘levamisole-sensitive’ nAChR, L-AChR, [32]. If chronic nicotine increases sympathetic activity, this could stimulate Na,K-ATPase signaling pathways via PKA and cAMP, which also increases PLM phosphorylation. In cardiac myocytes, activation of PKA stimulates the activity of both α1 and α2 isoforms [11]. Alternatively, systemic nicotine might modulate ACh release or CGRP release from nerve terminals [33]. The possibility that chronic nicotine may also alter other systemic or presynaptic pathways cannot be excluded [34]. It will be important for future studies to identify all of the signaling partners in this important regulatory complex, and to define the specific interactions of PLM with the different Na,K-ATPase isoforms.

### Pharmacologic Implications

The steady depolarization produced by chronic nicotine is expected to lower the safety factor for neuromuscular transmission by reducing muscle excitability. While there is normally a large safety factor for neuromuscular transmission (EPP amplitude about 15–20 mV above the threshold for triggering an action potential), any depolarization will lower this margin and inactivate Na^+^ channels, which are present at the postsynaptic neuromuscular junction at 20-fold higher density than on non-junctional sarcolemma. The consequences of a more depolarized end plate will be greatest under conditions in which neuromuscular transmission is already compromised. This occurs, for example, during intense exercise when high-frequency nerve activity depolarizes the resting potential, and in neuromuscular disorders such as myasthenia gravis in which the safety factor is already low [35].

**In summary**, chronic nicotine exposure alters Na,K-ATPase activity in skeletal muscle by interacting with the nAChR-Na,K-ATPase-PLM complex. This regulatory complex is capable of functional interactions with both Na,K-ATPase α1 and α2 isoforms, to both stimulate and inhibit enzyme activity. The same nicotine treatment activates PKCα/β2 and PKCδ and promotes phosphorylation of PLM at Ser^63^ and Ser^68^, supporting the idea that the functional interaction between the nAChR and the Na,K-ATPase may be mediated by this mechanism.
